# Clinical efficacy of amniotic membrane with biphasic calcium phosphate in guided tissue regeneration of intrabony defects- a randomized controlled clinical trial

**DOI:** 10.1186/s40824-021-00217-7

**Published:** 2021-05-06

**Authors:** Nivedha Venkatesan, Vamsi Lavu, S. K. Balaji

**Affiliations:** Department of Periodontology, Sri Ramachandra Institute of Higher Education and Research, Chennai, India

**Keywords:** Alveolar bone loss, Amniotic membrane, Chronic periodontitis, Guided tissue regeneration, Randomized Controlled Trial

## Abstract

**Background:**

The concept of periodontal regeneration has been revolutionised since the introduction of growth factors and bioactive bone substitutes which ensures optimal regeneration of the diseased periodontium. The aim of the present study was to evaluate the efficacy of Amniotic membrane + Biphasic Calcium phosphate as compared to Collagen membrane + Biphasic Calcium phosphate for the management of periodontal intrabony defects.

**Methods:**

50 systemically healthy patients with localised moderate to severe periodontitis, sites which had a Probing Pocket Depth (PPD) ≥ 6 mm and an intrabony component of ≥ 3 mm as detected on Intra oral periapical radiographs (IOPAR) and bone sounding were recruited based on specific inclusion and exclusion criteria. They were randomly allocated by computer generated tables to Collagen membrane + Biphasic Calcium phosphate and Amniotic membrane + Biphasic Calcium phosphate groups. The amount of bone fill and changes in Probing Pocket Depth, Clinical Attachment Level were measured at baseline and six months.

**Results:**

The results of the present study showed a mean reduction in the PPD of 2.89 ± 0.69 mm in the Collagen membrane + Biphasic Calcium phosphate group and 2.95 ± 0.57 mm in the Amniotic membrane + Biphasic Calcium phosphate group and CAL gain of 2.60 ± 1.43 mm in Collagen membrane + Biphasic Calcium phosphate group 3.18 ± 1.13 mm in the Amniotic membrane + Biphasic Calcium phosphate group at 6 months follow-up with no statistical significance between the groups. In terms of Defect resolution, 98.62 ± 6.51 % was achieved in Collagen membrane + Biphasic Calcium phosphate group and 98.25 ± 7.21 % in Amniotic membrane + Biphasic Calcium phosphate group.

**Conclusions:**

Within the limitations of the present study, it can be concluded that AM can be used as a barrier membrane, in conjunction with Biphasic calcium phosphate, and provides comparable results to Collagen membrane with Biphasic calcium phosphate when used in the management of periodontal intrabony defects.

**Supplementary Information:**

The online version contains supplementary material available at 10.1186/s40824-021-00217-7.

## Background

The last two decades have witnessed significant progress in the various facets of periodontology including biomaterials used to achieve periodontal regeneration. With the advent of tissue engineering, the regeneration of the periodontium is a more active therapeutic modality than in the past. The tissue engineering approach involves combining mechanical, cellular aspects with biologic mediators to facilitate reconstruction/regeneration of a particular tissue [1]. Anton Sculean, in 2017 put forth clinical protocols, which have shown to enhance periodontal regeneration and clinical outcomes in periodontal intrabony and class II furcation defects. These include: (a) Use of Enamel Matrix Proteins (b) Guided Tissue Regeneration (c) Use of bone grafts enriched with growth factors (Or) Combination therapy [2].

Guided tissue regeneration with barrier membranes has been demonstrated to be effective in preventing epithelial and gingival connective tissue cells from migrating onto the instrumented root surface [3]. The primary outcomes in the treatment of intrabony defects achieved by guided tissue regeneration are (i) increase in functional tooth support (clinical attachment and bone levels); (ii) reduction in pocket depth; and (iii) minimal gingival recession [4].

Amongst the biomaterials used as barrier membranes, Type I collagen (from varied sources) have been widely used in guided tissue regeneration procedures [5]. The Type 1 collagen is an ideal biomaterial as it is a extracellular macromolecule of the periodontal connective tissue, which aids in fibroblast chemotaxis, epithelial cell inhibition and has weak immunogenicity [6]. Combining collagen membrane with bone substitutes prevents soft tissue collapse, especially in non-contained infrabony defects, and thus ensure space maintenance [5]. However, collagen membrane act mainly as barriers, and they are considered biologically inactive. Fetal membranes as biodegradable materials have been employed in medicine for decades. Human amniotic membrane (hAM), the innermost layer of fetal membranes, was first used for transplantation of skin in 1910 [7]. hAM serves as a fibrillar scaffold and has been reported to serve as a reservoir of stem cells, modulates angiogenesis and promote wound healing [7]. hAM is composed of 5 layers: a single layer of epithelium, basement membrane, compact layer, fibroblast layer, and spongy layer [8]. The basement membrane is made up of collagen Types IV and VII, fibronectin, laminin, and hyaluronic acid secreted by the epithelial layer [9]. The compact layer is made up of collagen Types I and III synthesized by mesenchymal stem cells, whereas the fibroblast layer contains macrophages [8]. The presence of the non collagenous proteins in the hAM makes it superior to the collagen membrane as these proteins serve to modulate the cell behavior in a healing wound. This has been proven in vitro, through the culture of the human periodontal ligament fibroblasts using human amniotic membranes as scaffolds. The authors reported on cell viability and proliferation of the human periodontal ligament fibroblasts, with a significant increase in proliferation between Day 1–7 and significant decrease thereafter [9].

In an attempt to further improve the clinical outcomes of GTR techniques, particularly in unfavourable and large defects, it has been suggested combining the use of bone grafts with the barrier membrane to obtain a synergistic effect termed as Combined Periodontal Regenerative Therapy (CPRT) [10]. This approach leads to the assemblage of different regenerative principles, such as osteoconductivity and osteoinductivity provided by the bone grafts and space provision, wound stability provided by the barrier membranes. In this regard, a recent clinical trial evaluated the clinical, anti-inflammatory and anti-infective properties of the human amniotic membrane when used with bone graft for the periodontal regeneration. The authors in this trial reported, the use of the combination of amniotic membrane with bone graft versus bone graft alone (control) resulted in improved pocket depth reduction, attachment gain and reduction in Interleukin 1 beta levels in the Gingival crevicular fluid in the amniotic membrane group [11].

A comparison of the clinical healing outcomes when using collagen membrane versus amniotic membrane is the need of the hour. Hence, the present clinical trial is performed to test the equivalence hypothesis : the use of amniotic membrane with bone graft is equally effective as collagen membrane with bone graft in regeneration of a periodontal intrabony defect at 6 months follow up.

## Materials and methods

### Trial design

The study was designed as a prospective double-blinded randomized controlled clinical trial with two arm parallel group design and fulfilled the CONSORT guidelines. Patients visiting the out-patient department of Periodontology & Implantology, Faculty of Dental Sciences, Sri Ramachandra Institute of Higher Education and Research (SRIHER), Chennai, India fulfilling specific inclusion and exclusion criteria were enrolled in the study after obtaining informed consent. The study protocol was approved by the Institutional Ethics Committee of Sri Ramachandra Institute of Higher Education and Research (IEC/18/DEC/145/51) and was registered in the Clinical Trial Registry of India [Ref No: CTRI/2020/03/0240075].

### Participant selection

Patients of age 21–50 years with moderate to severe periodontitis, sites which had a Probing Pocket Depth (PPD) of ≥ 6 mm and an intrabony component of ≥ 3 mm as detected on Intra oral periapical radiographs (IOPAR) and bone sounding (Sali [12]) were included. Intrabony defect angle of < 40 degree (Sali [12]) including Circumferential defect with interdental intrabony defect depth of > 3mm were included. Exclusion Criterias were; Current Smokers (smokers who have smoked >/=100 cigarettes in their lifetime and currently smoke) (CDC 2017). Teeth with intrabony defect component and furcation involvement, systemic chronic conditions known to be related to periodontitis and other conditions that can influence systemic inflammation, pregnant / lactating mothers, and previous history of periodontal treatment at the selected site were excluded from the study.

### Sample size calculation

The sample size [12] was calculated with an assumed power of 80 % (β = 0.89) to detect a minimum clinically significant difference in probing depth of 1 mm and a standard deviation of 2.1 and alpha error (5 %), a total of 40 (20 per group) patients were required and recruited in the following study. Allowing for dropouts, a total of 50 patients were recruited (25 in each group).

### Randomization

**Fig. 1:**
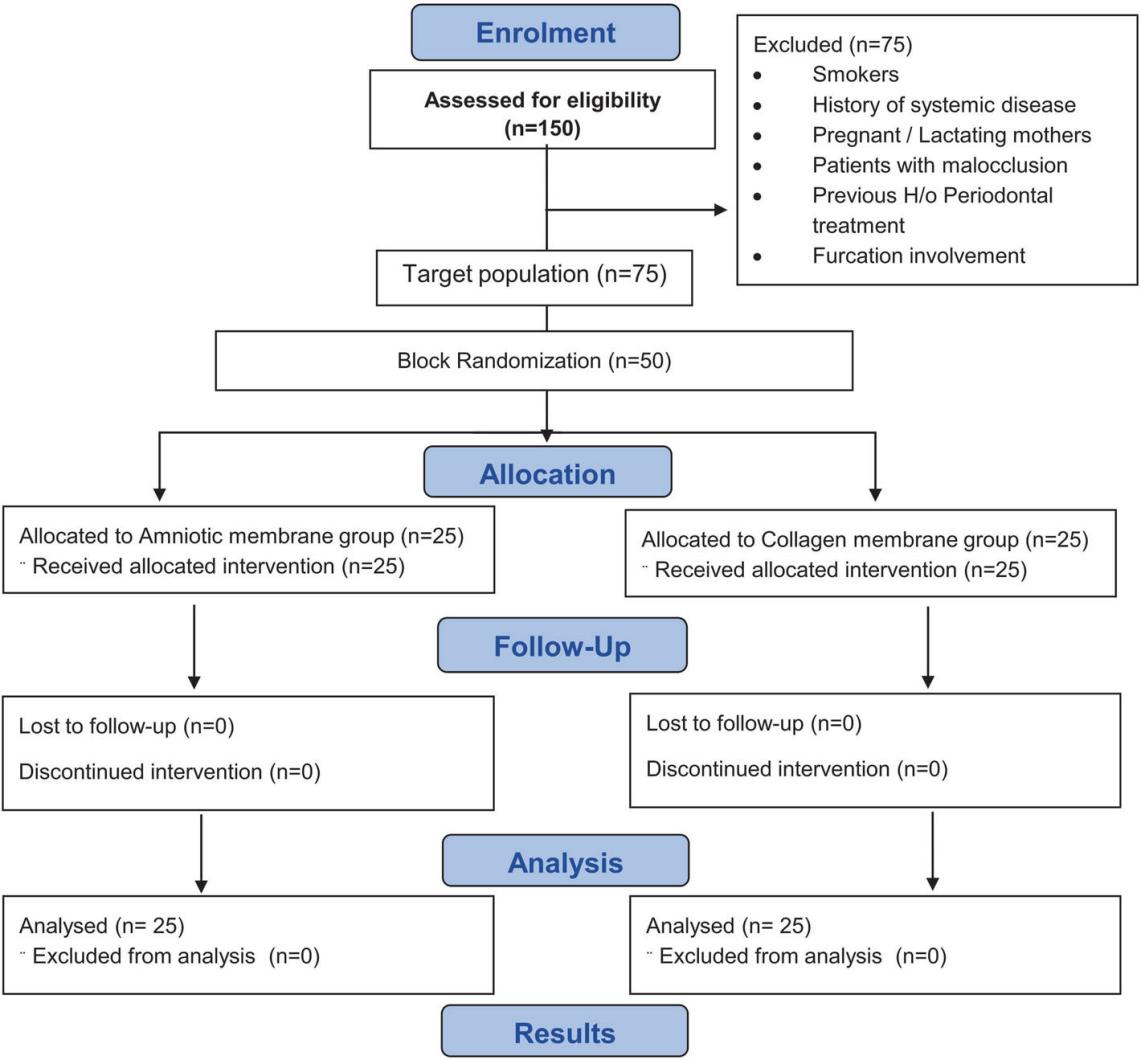
CONSORT flow chart of the present study

A random number table was used to select 50 subjects for intervention from the target population of 75 and selected subjects were allocated into blocks containing 4 subjects per block. Figure 1 shows the CONSORT chart for the present study. In each block, randomisation of the intervention procedures was done using the following code of 1221; 1122; 2112; 1212; 2121, (wherein 1- represents Collagen membrane + Biphasic alloplastic bone substitute (Control) 2- represents Amniotic membrane + Biphasic alloplastic bone substitute (Test)).

### Allocation concealment & blinding

Allocation concealment was done by placing the interventions in a sealed envelope which was opened by the surgeon prior to start of the surgery. Patients and statistician were blinded to the intervention.

### Outcome variables

The primary outcome variables included the assessment of change in Probing Pocket Depth (PPD)/ Clinical Attachment Level (CAL) and Radiographic bone fill. Secondary outcome variables included Visual Analogue Scale (VAS) [13] & Wound Healing Index [14].

### Pre‐surgical preparation

Surgical guides were fabricated with cold cure resin on cast models of patients, obtained by alginate impressions. The guides covered the occlusal surface of the tooth being treated including at least one tooth mesial and distal to it, extending both buccal and lingual/ palatal. A groove was made in the guide with a fissure bur extending in an apico-coronal direction at the point where probing pocket depth (PPD) and infra-bony defect were identified. The groove serves to provide reproducible alignment of the probe (Hu-Friedy PCP-12) during pre-op and post-op measurements. All clinical parameters were analysed by a single trained examiner (NV).

Clinical baseline and 6 month follow-up measurements, Probing Pocket Depth (PPD), Clinical Attachment Loss (CAL), were recorded by taking CEJ as the reference landmark and aligning the probe through the groove in the surgical guide, which ensures proper reproducible angulation.

Each patient involved in the study received a full diagnostic workup, which included Intra -oral periapical radiograph of the affected site, study casts and intraoral photographs. A thorough sub-gingival scaling was performed along with possible elimination of the etiological factors and oral hygiene instructions were given. Routine Blood parameters were checked pre-operatively (FBS/PPBS, BT, CT, CBC, INR). After oral prophylaxis, root planing was done under local anaesthesia using Gracey’s Area specific curettes. Occlusal adjustments were addressed when indicated. Approximately 3–4 weeks following the nonsurgical periodontal therapy, patients with persistent periodontal pockets (> 5mm) were taken up for further periodontal flap surgery.

0.12 % of 10ml of chlorhexidine mouth rinse was given to the patient as a preoperative preparation, where the patient was asked to swish for 30 s and then expectorated / suctioned to decrease the bacterial load prior to the start of the surgery.

### Surgical intervention

All surgical procedures were performed by the same expert operator (VL) with a longstanding clinical experience (> 10 years) in periodontal surgery.

**Fig. 2:**
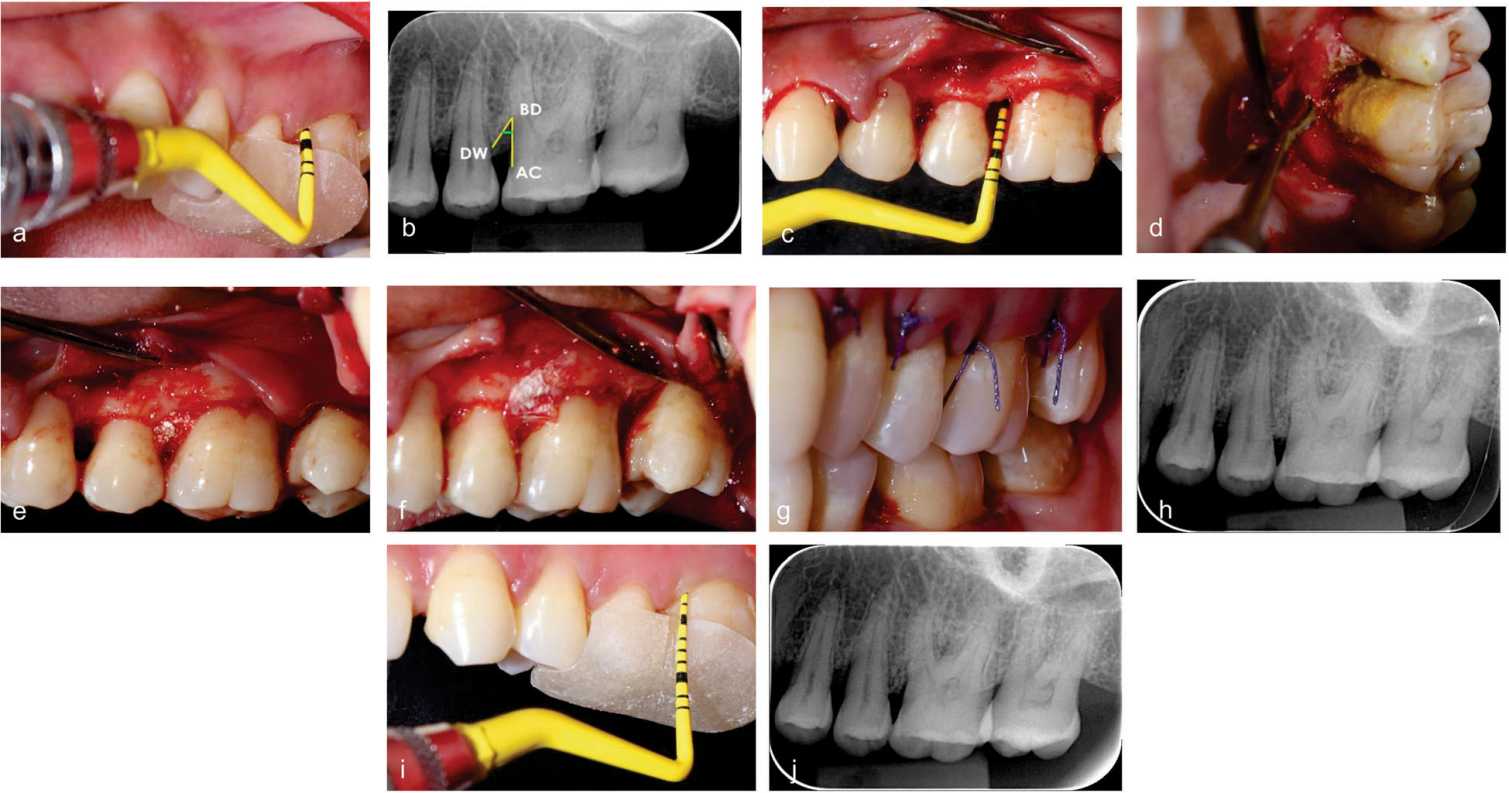
**a** Pre-operative clinical images with acrylic stent in place to measure the PPD, CAL in the region of mesiobuccal [8(9) mm]. **b** Preoperative digital intra oral radiograph with intrabony defect in relation to mesial aspect of 26. **c** Simplified papilla preservation done in relation to mesial of 26 and flaps raised, Intra-operative clinical measurement of the two wall defect from the CEJ to the base of the defect in relation to mesiobuccal aspect of 26 = 5mm. **d** Root conditioning done with tetracycline in relation to 26. **e** Defect site grafted with B-ostIN HT (HA60 %:BTCP40 %). **f** Amniotic membrane placed over the grafted site in relation to mesial aspect of 26. **g** External vertical mattress suturing done with 4 − 0 vicryl sutures. **h** Immediate post-operative digital intra oral radiograph showing complete defect fill in relation to mesial aspect of 26. **i** Six months post-operative clinical images with acrylic stent in place to measure the PPD, CAL in the region of mesiobuccal [2(3) mm]. **j** Six months post-operative RVG in relation to mesial aspect of 26 showing complete defect fill

**Fig. 3:**
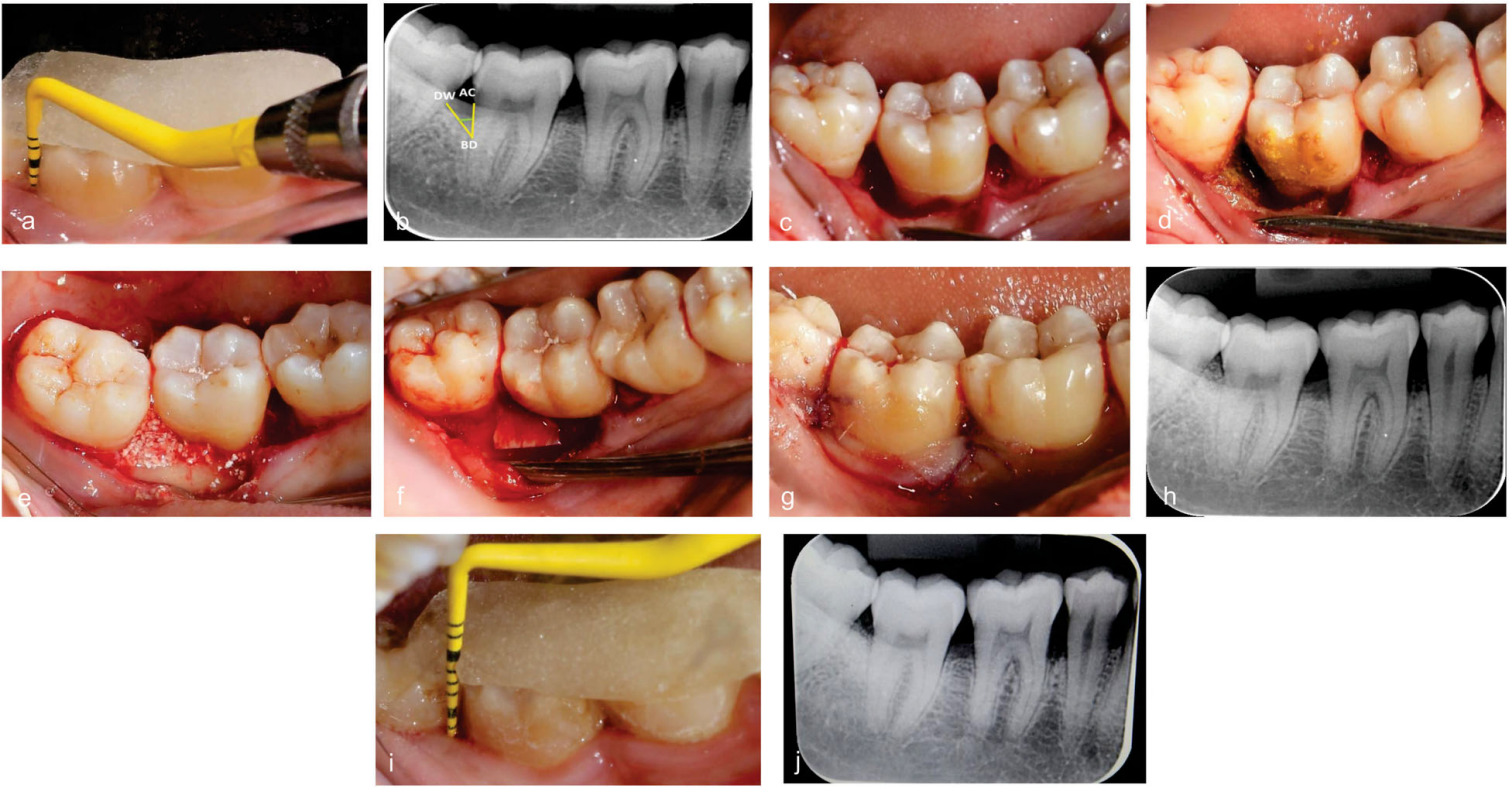
**a** Pre-operative clinical images with acrylic stent images with acrylic stent in place to measure the PPD, CAL in the region of distobuccal [7(7) mm] aspect of 47. **b** Preoperative RVG with intrabony defect in relation to distal aspect of 47. **c** Full thickness flap elevated in relation to 45, 46, 47 with simplified papilla preservation in relation to distal of 47 showing two wall defect from the CEJ to the base of the defect in relation to distobuccal aspect of 47 = 6mm. **d** Root conditioning done with tetracycline in relation to 47. **e** Defect site grafted with B-ostin HT(HA60 %:BTCP40 %) and (**f**) collagen membrane placed over the grafted area in relation to 47. **g** External vertical mattress suturing done with 4 − 0 vicryl sutures. **h** Immediate post-operative digital intra oral radiograph showing complete defect fill in relation to distal aspect of 47. **i** Six months post-operative clinical images with acrylic stent in place to measure the PPD, CAL in the region of distobuccal [2(3) mm] aspect of 47. **j** Six months post-operative digital intra oral radiograph in relation to distal aspect of 47 showing complete defect fill

Under local anaesthesia, simplified papilla preservation flap [15] was performed. A Swann Morton No.63 microsurgical blade was used, the first incision was given across the defect associated papilla in an oblique direction beneath the contact point and was carried out keeping the blade parallel to the long axis of the teeth to avoid excessive thinning of the remaining interdental tissues following which a full thickness mucoperiosteal flap was elevated exposing the intrabony defect. After which, defect debridement and root planing was performed using Gracey’s area specific curettes. Root conditioning was done using Tetracycline Hydrochloride 5 % solution and decortication of the defect site was done with the low speed micromotor. The defect was filled with biphasic alloplastic bone substitute material [(60 % hydroxyapatite: 40 % Beta TCP) (B-OstIN HT -0.5 to 1mm particle size), Basic Healthcare Products Pvt Ltd, India]. Following grafting of the defect site, it was followed by placement of Amniotic membrane [sourced from Tissue Bank-ACTREC, Tata Memorial Center, India] (Fig. 2a-j) or Collagen membrane [Ossix Plus membrane, Dentsply Sirona, USA] (Fig. 3a-j). The flaps were then trimmed and approximated by external vertical mattress using 4 − 0 vicryl resorbable sutures and Cyanoacrylate dressing was placed over the surgical site.

### Post‐surgical care

Each patient was prescribed analgesics & antibiotics after surgery. Antibiotic Cap. Amoxcillin 500 mg thrice daily and analgesic T. Aciclofenac + Paracetamol combinations was prescribed twice daily for three days following the surgery. A 60-second rinse with 0.12 % CHX was prescribed 3 times/day for first 2 weeks. Patients were recalled a1 3, 10 days, 1, 3 & 6 months for professional oral hygiene maintenance and data recording.

### Statistical analysis

Statistical analysis was performed with SPSS 20 software. Descriptive statistics were recorded as mean values, standard deviations, frequencies and percentages. Analysis was done at patient and technique levels. The primary outcome variable was change in Probing Pocket Depth (PPD) Clinical Attachment Level (CAL) and Radiographic bone fill. Secondary variables included assessment of VAS and WHI scores. The significance of the differences between the groups was evaluated using T-Test. P value < 0.005 was considered significant.

## Results

Fifty patients (22 males and 28 females) were included in the study. The patients were equally distributed within the AM + BiCP and CM + BiCP groups in terms of demographic characteristics and clinical parameters assessed at baseline (Probing depth/ Clinical Attachment Level and Defect angle) and distribution of the defect walls within AM + BiCP and CM + BiCP (Table 1).

**Table 1:** Demographic data of the study population with pre-operative clinical parameters (Analysis by t-test square test)

	CM + BiCP (Mean ± Std)	AM + BiCP (Mean ± Std)	*p* value
AGE (years)	34.84 ± 7.13	34.72 ± 6.11	0.948
OHI-S	1.60 ± 0.40	1.83 ± 0.48	0.083
PPD (mm)	7.36 ± 0.81	7.24 ± 1.01	0.611
CAL (mm)	7.32 ± 0.09	7.32 ± 1.02	0.500
DEFECT ANGLE (º)	30.02 ± 3.55	31.06 ± 5.17	0.237
**Distribution of Defect Walls within control and test group**			
	**One wall**	**Two wall**	**Combined defect**
CM + BiCP	10 (40.0 %)	10(40.0 %)	5 (20.0 %)
AM + BiCP	11 (44.0 %)	8 (32.0 %)	6 (24.0 %)

The results of the present study showed a mean reduction in the PPD of 2.89 ± 0.69 mm in the CM + BiCP group and 2.95 ± 0.57 mm in the AM + BiCP group and CAL gain of 2.60 ± 1.43 mm in CM + BiCP group, 3.18 ± 1.13 mm in the AM + BiCP group at 6 months follow-up with no statistical significance between the groups. In terms of Defect resolution, 98.62 ± 6.51 % was achieved in CM + BiCP group and 98.25 ± 7.21 % in AM + BiCP group (Table 2).

**Table 2:** Intergroup Analysis Of Primary Outcome Variables (PPD,CAL, Defect Resolution) and Secondary Outcome Variables (VAS & WHI) at day 3 and day 10 follow-up between AM + BiCP and CM + BiCP groups at 6 months follow-up (Analysis by - Student t-test)

	CM + BiCP (Mean ± Std)	AM + BiCP (Mean ± Std)	*p* value
**PRIMARY OUTCOME VARIABLES**			
PPD (mm)	2.89 ± 0.69	2.95 ± 0.57	0.346
CAL (mm)	2.60 ± 1.43	3.18 ± 1.13	0.054
DEFECT RESOLUTION (%)	98.62 ± 6.51 %	98.25 ± 7.21 %	0.950
**SECONDARY OUTCOME VARIABLES**			
DAY 3 WHI	3.36 ± 0.48	3.44 ± 0.65	0.313
DAY 10 WHI	3.80 ± 0.75	4.12 ± 0.43	0.014
DAY 3 VAS	2.60 ± 0.57	2.04 ± 0.88	0.190
DAY 10 VAS	1.08 ± 0.75	0.92 ± 0.75	0.007

Secondary outcome variables measured were Visual Analog Scale (VAS) and Wound Healing Index (WHI) at Day 3 and Day 10 follow-up. Higher WHI scores and reduced VAS scores were observed in the AM + BiCP group than the CM + BiCP at day 3 and day 10 follow-up, however no statistical significance were observed between the two groups (Table 2).

## Discussion

The present randomized controlled clinical trial compared clinical/ radiological and patient centered outcomes following the application of Amniotic membrane/Collagen membrane, (porcine derived) in combination with Biphasic Calcium phosphate, (60 % Hydroxyapatitie: 40 % ß TCP) for the surgical reconstruction of periodontal intrabony defects.

The two surgical groups were compared in terms of mean differences in Probing Pocket Depth, Clinical Attachment Level and radiographic bone fill at 6 months follow-up. Patient centered outcomes in terms of Visual Analog Scale and Wound Healing Index were assessed at the 3 and 10 day follow-up of the surgical procedure. The types of bony defects included in the present study varied from three-walled to one-walled intrabony defects. It is noteworthy that most defects were also combined. It has been reported that three-walled defects show more predictable results after GTR procedures compared to combined or complex defects. However, the results of the present study revealed that morphologic variations of the bony defects did not influence the efficacy of GTR treatment in either group.

To date, there are no published data on the clinical use and assessment of patient centered outcome measures of AM alone or in conjunction with biphasic alloplastic graft material for the treatment of intrabony defects. The results of the present study showed a mean reduction in the PPD of 2.89 ± 0.69 mm in the CM + BiCP group and 2.95 ± 0.57 mm in the AM + BiCP group and CAL gain of 2.60 ± 1.43 mm in CM + BiCP group 3.18 ± 1.13 mm in the AM + BiCP group at 6 months follow-up. Although the reduction in the pocket depth and the gain in clinical attachment (clinical measures indicative of the healing) was better in the amniotic membrane with bone graft group as compared to collagen membrane with bone graft group, a statistical significance could not be established The above mentioned findings are in concurrence with the observations in systematic reviews by Laurell et al., [16] and Parrish LC et al., [17] wherein the authors reported the average CAL gain after treating periodontal intrabony defects with biodegradable membranes with and without graft material was 2.96 mm and 3.50 mm respectively.

Collagen membranes are the most commonly employed barrier membranes for GTR, as they facilitate migration of periodontal ligament fibroblasts and provide an early scaffold for neo-angiogenesis. However, these types of membranes act mainly as barriers, and they are considered biologically inactive, while owing to the unique characteristics of AM, it acts as a biological membrane, providing wound protection and bacteriostatic effect [18].

As described by Schultz et al. [19], dynamic reciprocity is an ongoing, bidirectional interaction among cells and their surrounding microenvironment. These interactions take several forms that may be categorized as direct or indirect. AM offers a scaffold for proliferation and differentiation owing to the presence of elastin, nidogen, collagen types I, III, IV, V, and VI, elastin, and hyaluronic acid. AM contains growth factors such as platelet-derived growth factors alpha and beta and transforming growth factor beta that hasten formation of granulation tissue by stimulating the growth of fibroblasts and stimulates neovascularization [20]. Laminin-5 in amniotic membrane has a high affinity to gingival epithelial cells, producing an early physiologic seal with the wound surface and thereby facilitating cell migration, accelerated wound healing [21]. AM also contains Fibronectin which acts as the recognition system that guides cell positioning [22]. Fibronectin is a matrix protein which contributes to cell migration and attachment through the RGD sequence [23]. One of the major advantages of AM, in comparison to other bio-degradable membranes, is its thickness (320 μm) and good adaptability which owes to the increased adaptability of the membrane to the defect morphology [24].

Randomised controlled trial have been performed by several authors [12, 25, 26] comparing AM/Biogide in combination with allograft and xenograft material for the management of grade II furcation defects and intrabony defects. After a post-operative period of 6 and 9 months, both the test and control groups showed significant reduction in PPD, CAL, and increased in percentage of bone fill, without any significant differences between the groups assessed.

The property of degradability of barrier membrane will influence the surgical outcome of GTR. Porcine- derived collagen membranes in GTR procedures have been shown to resorb within 4 to 6 months [27]. The degradation period of amniotic membrane can be hypothesised from studies of wound dressing and bladder reconstruction [28], it can be put forth that placement of AM as barrier membrane beneath a periodontal flap, prevents the early exposure of the surgical site. The membrane shows reduction in structural stability by 14-21st day as a result of mucoid degeneration [29, 30]. The gains in CAL and reductions in PPD in the present study make it safe to speculate that the absorption of AM was slow enough to produce the desired effects.

In an attempt to further improve the clinical outcomes of GTR, the present study was designed to employ a combined periodontal regenerative technique. The study included one wall, two wall, three wall and combined osseous defects with an intrabony defect angle of ≤ 40°. Owing to the bioactive properties and chemical similarity to the mineral phase of bone, calcium phosphate based biomaterials (CaP) are widely used for bone regeneration [31].

By modifying the HA/β-TCP ratio and, thus, the solubility of ceramic, it is possible to influence the pattern of resorption. On comparison with different ratios of HA and β-TCP (HA100; 80:20; 60:40) in a dog model, the 60:40 group showed more new bone formation and less residual bone mineral remaining at the end of 24 weeks [32, 33]. In the present study we have used Biphasic calcium phosphate in the ratio of 60 % HA: 40 % β-TCP which would provide the same degree of osteoconductive property in both the intervention groups. The bone graft used had a porosity of 60–70 % and a pore size of 100–300 micrometers which allows for optimal revascularization and bone regeneration. In addition, the particle size used was 0.5 to 1mm which supplements the defect fill and the osteoconductive ability of the bone graft.

Surgical access to the intrabony defects was selected from three different surgical approaches: the simplified papilla preservation flap; the modified papilla preservation technique; and the crestal incision based on the available interdental space. The degree of wounding and flap reflection are minimised by these techniques and ensures wound stability, primary closure, and space maintenance [34].

Cortellini P et al., [35], in a multicenter randomized clinical trial, evaluated the outcomes of the simplified papilla preservation flap technique used with barrier membranes for intrabony defect management and reported favourable clinical outcomes and lesser post operative morbidity. Hence, in the present study we have performed the simplified papilla preservation technique to gain access to the periodontal intrabony defects.

Percentage of bone fill at the end six months of the surgical procedure was assessed in terms of Defect depth reduction. The present study utilised standardised radiography to diminish the variations of the projection geometry between pre- and post-surgical radiographs using the long cone parallel technique with a commercially available positioning guide and the radiographic analysis was done by means of computer aided program. (Image J software). Defect depth reduction in the present study at 6 months follow-up in CM + BiCP and AM + BiCP groups were 98.62 ± 6.51 % and 98.25 ± 7.21 % respectively with no statistical significance (*p* < 0.950).

At present there are no clinical studies evaluating the Patient Centered Outcome measures and wound healing following the Collagen membrane and Amniotic membrane in the management of periodontal intrabony defects. AAP Consensus report on Periodontal regeneration of intrabony defects, 2015 [36] has concluded that, in future research designs, objective measures of postoperative pain evaluated in real time should be incorporated, inclusion of modulating factors such as treatment modality, operator experience, surgical technique, and patient-related factors. The patient centered outcomes were evaluated using Visual Analog Scale for pain at Day 3 and 10. The mean VAS score at Day 3 were 2.04 ± 0.88 and 2.60 ± 0.57 in the AM + BiCP and CM + BiCP groups respectively with no statistical difference between them (*P* value < 0.190).

Post-operative healing was evaluated using the Wound Healing Index [14]. Wound healing after the surgical procedure was assessed (3 days, 10 days) and was quantified and graded. In the present study, the mean WHI scores at day 10 following the surgery were 3.80 ± 0.75 and 4.12 ± 0.43 for CM + BiCP and AM + BiCP groups respectively with no statistical difference (P value < 0.014). However, in one of the cases in the CM + BiCP group, on day 10 follow-up, there was membrane exposure (with no sign of infection) observed at the surgical site which subsequently healed (following removal of exposed portion of the membrane) allowing for no further complications in regeneration. No post-operative complications were observed during the early healing phase in the AM + BiCP group, owing to the easy adaptability of the amniotic membrane over the defect site providing an early physiologic seal.

A limitation of this study was the use of 2D imaging analysis with the help of Radiovisuography (RVG) instead of 3D analysis with CBCT. Re-entry was not considered, as none of the teeth included in this study were candidates for extraction, a histologic study was not performed.

Guided tissue regeneration in the present era can no longer be considered as a single treatment approach. There is paramount of evidence available to consider GTR as a multifactorial treatment approach comprising careful selection of patients and defects, different surgical techniques, various types of membranes and adjunctive materials and many suturing approaches. All the cited components could be combined to build up different treatment strategies in order to increase the predictability of the treatment outcome.

## Conclusions

Within the limitations of the current study, it can be concluded that Amniotic membrane in conjunction with Biphasic calcium phosphate, appears to provide better patient related outcomes and comparable clinical and radiological outcomes as compared with porcine derived Collagen membrane and Biphasic calcium phosphate combination in the management of periodontal intrabony defects. However future studies, directed at overcoming the limitations of amniotic membrane which include the handling characteristics, structural stability and limited availability need to be performed.

## Supplementary Information

**Additional file 1: Supplementary Figure 1.** Schematic diagram of the changes in the clinical parameters -Probing Pocket Depth (PPD) and Clinical Attachment Level (CAL) as assessed in the periodontium between health and disease.

**Additional file 2: Supplementary Figure 2.** Schematic diagram of the procedure performed to achieve the outcome of periodontal regeneration.

## Data Availability

Available.

## Abbreviations

GTR: Guided Tissue Regeneration
AM: Amniotic Membrane
CM: Collagen Membrane
CPRT: Combined Periodontal Regenerative Therapy
PPD: Probing Pocket Depth
IOPAR: Intraoral Periapical Radiograpgh
CDC: Centre For Disease Control
CONSORT: Consolidated Standards of Reporting Trials
CAL: Clinical Attachment Level
WHI: Wound Healing Index
VAS: Visual Analog Scale
FBS: Fasting Blood Sugar
PPBS: Post Prandial Blood Sugar
BT: Bleeding Time
CT: Clotting Time.
CBC: Complete Blood Cell Count
INR: International Normalised Ratio
CEJ: Cemento- enamel Junction
HA: Hydroxyapatite
βTCP: Beta Tricalcium Phosphate
BiCP: Biphasic Calcium Phosphate
CHX: Chlorhexidine.
GCF: Gingival Crevicular Fluid
RGD: Arginine Glysine Aspartate
SPPT: Simplified Papilla Preservation Technique
AAP: American Association Of Periodontology
RVG: Radiovisiography
CBCT: Cone Beam Computer Tomography
2D: Two Dimensional
3D: Three Dimensional
