# Supplementary figures and images for: Clinical efficacy of amniotic membrane with biphasic calcium phosphate in guided tissue regeneration of intrabony defects- a randomized controlled clinical trial
Source: Biomater Res. 2021 May 6;25:15. doi: 10.1186/s40824-021-00217-7 (PMC8101164; doi:10.1186/s40824-021-00217-7)

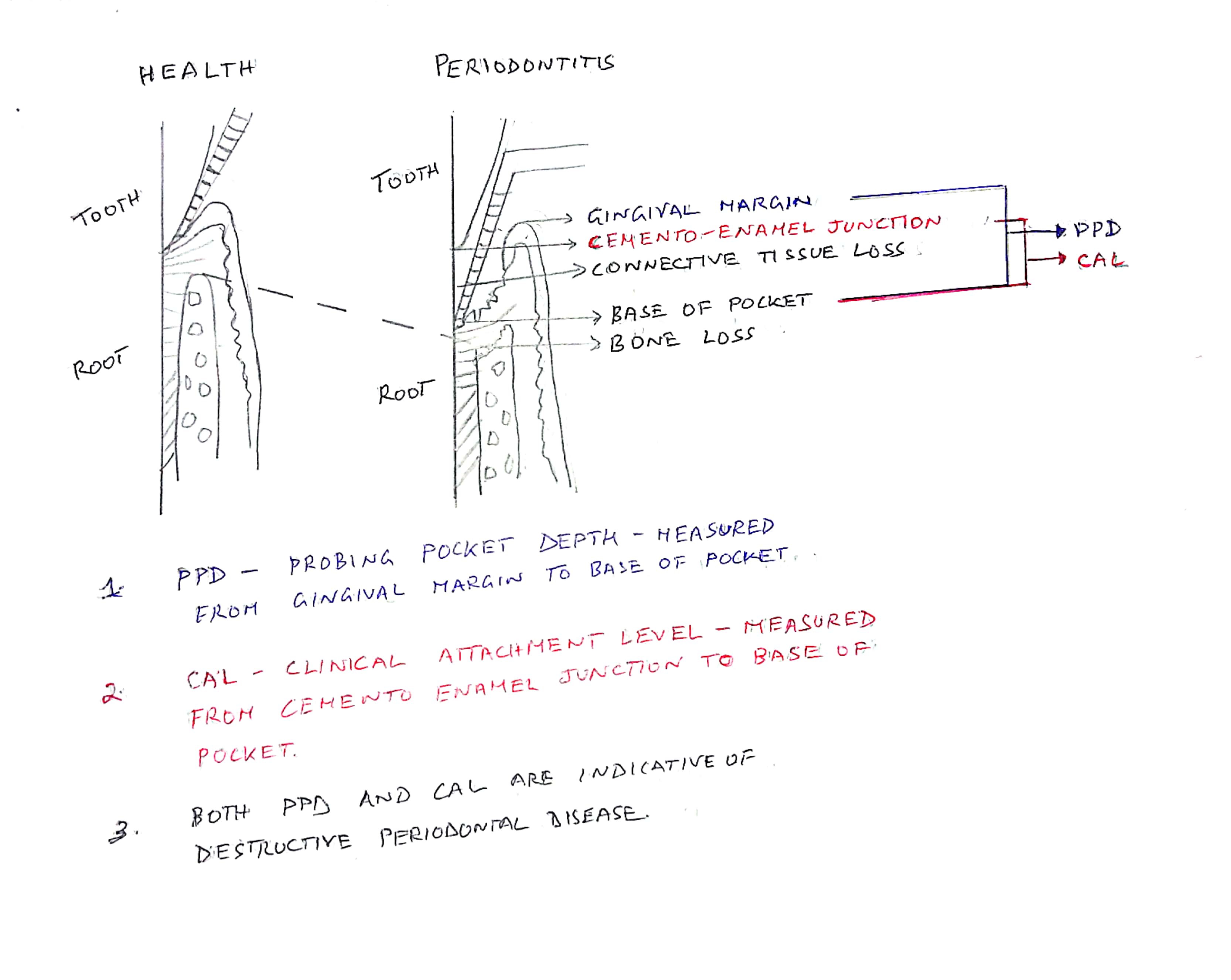

Supplement: Supplementary file 1 — Additional file 1: Supplementary Figure 1. Schematic diagram of the changes in the clinical parameters -Probing Pocket Depth (PPD) and Clinical Attachment Level (CAL) as assessed in the periodontium between health and disease. [file 40824_2021_217_MOESM1_ESM.jpg]

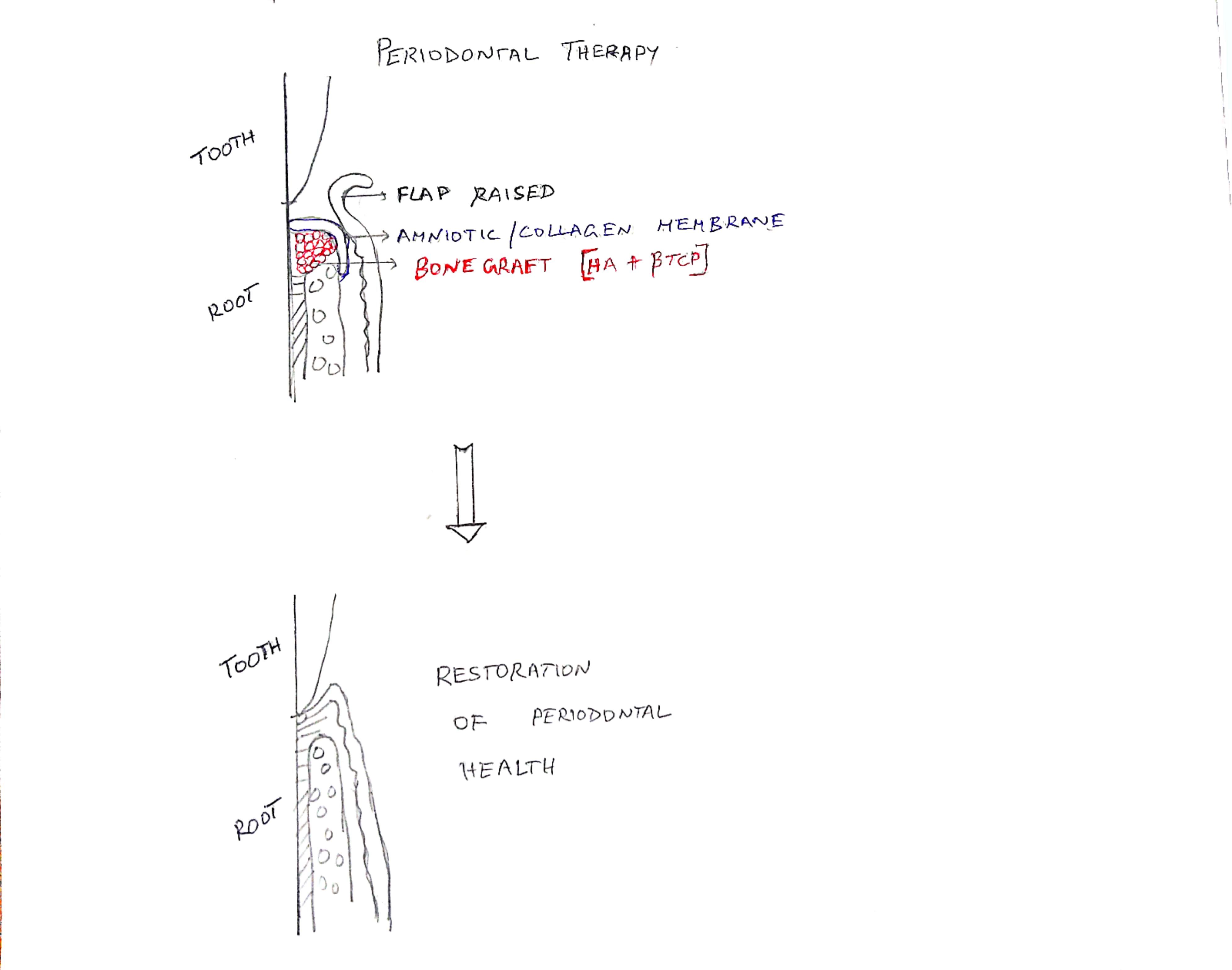

Supplement: Supplementary file 2 — Additional file 2: Supplementary Figure 2. Schematic diagram of the procedure performed to achieve the outcome of periodontal regeneration. [file 40824_2021_217_MOESM2_ESM.jpg]
